# Boundary-Guided Differential Attention: Enhancing Camouflaged Object Detection Accuracy

**DOI:** 10.3390/jimaging11110412

**Published:** 2025-11-14

**Authors:** Hongliang Zhang, Bolin Xu, Sanxin Jiang

**Affiliations:** College of Electronics and Information Engineering, Shanghai University of Electric Power, Shanghai 201306, China; zhang15678469551@163.com (H.Z.); 16621021581@163.com (B.X.)

**Keywords:** camouflaged object detection, attention mechanism, boundary detection, deep neural network

## Abstract

Camouflaged Object Detection (COD) is a challenging computer vision task aimed at accurately identifying and segmenting objects seamlessly blended into their backgrounds. This task has broad applications across medical image segmentation, defect detection, agricultural image detection, security monitoring, and scientific research. Traditional COD methods often struggle with precise segmentation due to the high similarity between camouflaged objects and their surroundings. In this study, we introduce a Boundary-Guided Differential Attention Network (BDA-Net) to address these challenges. BDA-Net first extracts boundary features by fusing multi-scale image features and applying channel attention. Subsequently, it employs a differential attention mechanism, guided by these boundary features, to highlight camouflaged objects and suppress background information. The weighted features are then progressively fused to generate accurate camouflage object masks. Experimental results on the COD10K, NC4K, and CAMO datasets demonstrate that BDA-Net outperforms most state-of-the-art COD methods, achieving higher accuracy. Here we show that our approach improves detection accuracy by up to 3.6% on key metrics, offering a robust solution for precise camouflaged object segmentation.

## 1. Introduction

Camouflaged Object Detection (COD) is an emerging computer vision task aimed at accurately recognizing and segmenting camouflaged targets that are seamlessly hidden in their backgrounds [1]. COD has wide applications in various detection tasks across multiple domains, including medical image segmentation, defect detection, agricultural image detection (e.g., locust detection), security monitoring (e.g., obstacle detection), and scientific research (e.g., biological studies). Therefore, the first COD method based on deep neural networks [2] gained widespread attention immediately after its introduction.

Early COD methods [3], inspired by the hunting process of predators, typically included a search module for the preliminary localization of camouflaged objects, followed by a recognition module for accurate segmentation. Additionally, some approaches [4,5] drew inspiration from bionics, simulating the hunting and observation behaviors of animals, making COD simpler and more efficient. However, these methods remained somewhat rudimentary and struggled to segment camouflaged objects with precision.

To further improve performance, an increasing number of COD methods [6,7,8,9] developed in recent years have begun incorporating guidance information. These approaches either extract texture features from the input image or estimate boundary features of the camouflaged object, using them to guide the detection. Accurate boundary priors not only aid in locating camouflaged objects but also help mitigate boundary blurriness during object segmentation. However, existing methods often rely solely on low-level features from the input image for boundary extraction, overlooking the high-level features. Additionally, when it comes to object localization and segmentation, these methods tend to focus heavily on global information. Yet, due to the high similarity between camouflaged objects and their background, this global information often fails to effectively highlight the differences between the objects and their surroundings.

Currently, to better differentiate camouflaged objects from their backgrounds, numerous COD methods have introduced attention mechanisms. These attention mechanisms either focus on extracting boundary knowledge or enhancing the prominence of camouflaged objects. For example, the edge attention network [10] and edge-assisted position aware attention network [9] are designed to extract informative boundary features. Meanwhile, others, such as the overlapping window cross-layer attention mechanism [11,12], use high-level features to guide the enhancement of lower-level features, and the dual attention mechanism [13] captures the scale diversity of camouflaged objects. Methods like the parallel attention selection mechanism [14] and Multiple Attention Mechanisms (PAM) [15] use multiple attention mechanisms to emphasize the separation between background and camouflaged objects.

Inspired by how the human visual system achieves functional specialization and efficiency gains by responding to different frequency stimuli through distinct neural pathways, Liang et al. proposed the Efficient Frequency Injection Module (FIM). This module enhances the representational capacity of lightweight backbone networks by injecting fine-grained high-frequency features and object-level low-frequency features at different stages [16]. Liu et al. [17] propose a depth-aware attention fusion network that incorporates depth maps as auxiliary inputs to enhance the network’s perception of three-dimensional information. Concurrently, a ternary branch encoder is employed to extract color and depth information along with their interactive relationships. Guan et al. [18] proposed a dual-branch strategy to reconstruct structural and detailed features separately, addressing the disparity in reconstruction requirements between structure and detail. This approach aims to identify camouflaged objects and their edges. Zhang et al. [19] proposed collaborative cross-scale feature learning network(CCNet), which efficiently detects collaborative camouflaged targets by leveraging synergistic information between single images and camouflage image groups. Fang et al. [20] developed a method that uses learnable wavelets to extract high-frequency edge details, refines them by aggregating contextual features and sensing inter-branch differences, and employs a scene enhancement module with reverse attention to recover structural information from occluded areas. These specially designed attention mechanisms have significantly improved the accuracy of camouflaged object detection. However, few attention mechanisms are directly built to specifically focus on the distinction between camouflaged objects and their surroundings.

## 2. Motivation and Contributions

To address the dual challenges of accurately localizing camouflaged objects within complex backgrounds and suppressing distracting contextual information, this study introduces a Boundary-Guided Differential Attention Mechanism. This mechanism leverages the disparity between Global Average Pooling (GAP) and Global Max Pooling (GMP) to enhance subtle discriminative cues, thereby improving the model’s ability to distinguish camouflaged regions from the background.

**Figure 1:**
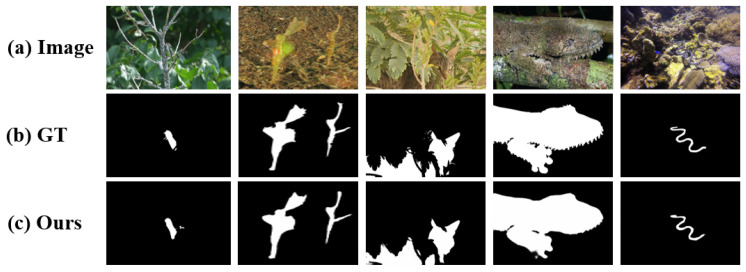
Detection results of camouflaged objects using BDA-Net. (**a**) Input images containing camouflaged objects; (**b**) Ground truth binary masks of the camouflaged objects; (**c**) Prediction results of the proposed BDA-Net.

Inspired by the hierarchical nature of human visual perception—where coarse boundary localization precedes fine-grained detail analysis—we propose the Boundary-Guided Differential Attention Network (BDA-Net) for camouflaged object detection.The visual detection results are shown in Figure 1. When boundary cues are available, human vision tends to disregard irrelevant background details and focus on object-specific features. Following this principle, BDA-Net first extracts boundary priors from multi-scale image features and then utilizes boundary-guided differential attention, derived from the GAP–GMP disparity, to suppress background responses and enhance object representations. The refined features are subsequently fused to produce accurate and detail-preserving segmentation of camouflaged targets.The main contributions of this work are as follows:

- Inspired by how the human eye perceives camouflaged objects, we propose a differential attention mechanism, which leverages the difference between GAP and GMP under the guidance of boundary knowledge to highlight camouflaged objects.
- We introduce a method for boundary prior extraction, which first fuses multi-scale features of the input image and then applies channel attention mechanism to emphasize boundary features.
- Building on these insights, we developed BDA-Net for COD. Guided by boundary priors, the network applies differential attention to the multi-scale features of the input image, achieving superior performance.

## 3. Method

### 3.1. Overall Architecture

**Figure 2:**
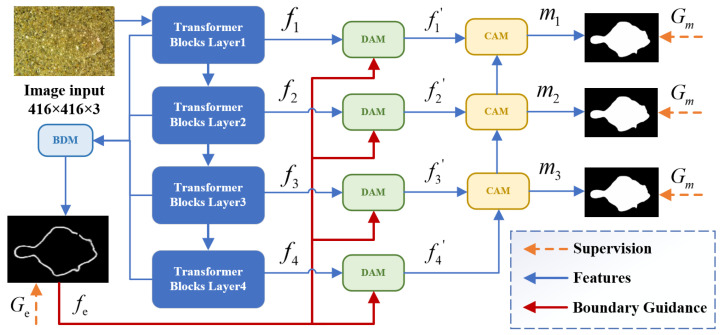
Overall architecture of BDA-Net. First, the input image, with a fixed resolution of 416×416×3, is processed by a Transformer-based backbone network to extract features at four different scales. These features are sent to the BDM module to extract boundary priors, which guide the differential attention mechanism. Simultaneously, the features are fed into four DAM modules, where the boundary priors help highlight the camouflaged object’s features. Finally, the CAM module fuses the enhanced features to produce the camouflage object mask.

The overall architecture of BDA-Net is illustrated in Figure 2. Broadly, BDA-Net consists of four main components: the PVTv2 backbone network for feature extraction, the Boundary Detection Module (BDM) for extracting boundary priors, the Differential Attention Module (DAM) for highlighting camouflaged objects, and the Context Aggregation Module (CAM) for predicting and segmenting the camouflaged objects.

In BDA-Net, the input image, with a fixed resolution of 416×416×3, is first processed by PVTv2, resulting in four multi-scale feature maps, denoted as $f_{i}\;\left(i=1,2,3,4\right)$. These features are processed in two ways: first, they are fed into the BDM to extract the boundary prior $f_{e}$, which serves as guidance for the differential attention mechanism; second, they are passed through the four DAM modules, where the boundary prior $f_{e}$ is used to highlight the camouflaged objects, producing refined feature maps $f_{i}^{\prime }\;\left(i=1,2,3,4\right)$. Finally, the three CAM modules progressively fuse the enhanced features to generate camouflaged object masks at three different scales, denoted as $m_{i}\;\left(i=1,2,3\right)$. Among them, $m_{1}$, which integrates all contextual information, has the highest precision and the largest size, and is chosen as the final output.

### 3.2. Boundary Detection Module

**Figure 3:**
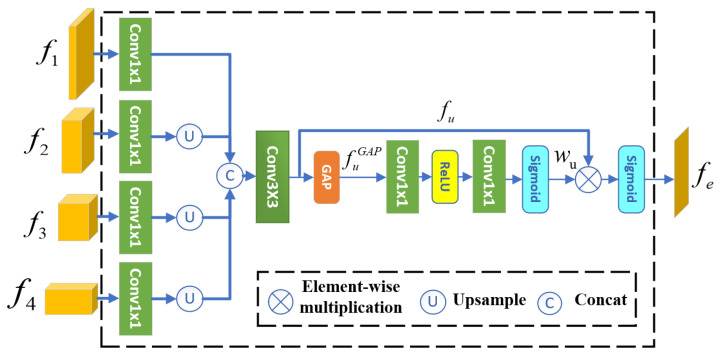
Architecture of the BDM module. This module first upsamples the smaller-scale features $f_{2}$, $f_{3}$, and $f_{4}$ to match the size of feature $f_{1}$. These features are then concatenated to form a single-scale feature map. Finally, a channel attention is applied to weight the feature map, highlighting the boundaries of the camouflaged object.

The architecture of BDM is shown in Figure 3. In the BDM, multi-scale features are first concatenated into a single-scale feature. Since the features $f_{1},f_{2},\cdots ,f_{4}$ correspond to four layers of the PVTv2 and have different dimensions, specifically 104×104, 52×52, ⋯, and 13×13, it’s necessary to upsample the smaller features $f_{2},f_{3}$, and $f_{4}$ to match the resolution of $f_{1}$. This ensures seamless concatenation, resulting in a unified feature map $f_{u}$ with a size of 104×104. A 3×3 convolution is then applied to this unified feature map, producing the fused feature $f_{u}$. It is important to note that a 1×1 convolution is applied to each feature before concatenation, primarily to adjust the number of channels. This process can be expressed by the following equation:

(1) $$f_{u}={Conv}_{3\times 3}\left(f_{1}+U\left(f_{2}\right)+U\left(f_{3}\right)+U\left(f_{4}\right)\right),$$

where U(·) represents the upsampling function. Since higher-level features typically capture the semantic information of the camouflaged object, while lower-level features focus more on its detailed structures, the simultaneous use of multi-scale features allows the BDM to be highly adaptive.

To enhance boundary features, we implemented a simplified version of the Efficient Channel Attention (ECA) [21] developed by Wang et al. Specifically, in ECA, the input features are duplicated into two copies. One copy is used to generate weights, which are then multiplied element-wise with the other copy. The resulting output is mapped to the range (0,1) using a Sigmoid function σ, forming the boundary prior.

To obtain the weights and extract boundary information, we apply GAP followed by a ReLU activation to the feature $f_{u}$. This not only reduces model parameters but also introduces non-linearity, enhancing the robustness of the BDM. Finally, the weights are constrained within the range of (0,1) using a Sigmoid function. The process can be expressed by the following equation:

(2) $$w_{e}=\sigma \left({Conv}_{1\times 1}\left(\mathrm{ReLU}\left({Conv}_{1\times 1}\left(f_{u}^{\mathrm{GAP}}\right)\right)\right)\right),$$

where $w_{e}$ and $f_{u}^{\mathrm{GAP}}$ represents the element weight and the features obtained by applying GAP to $f_{u}$, respectively.

### 3.3. Differential Attention Module

**Figure 4:**
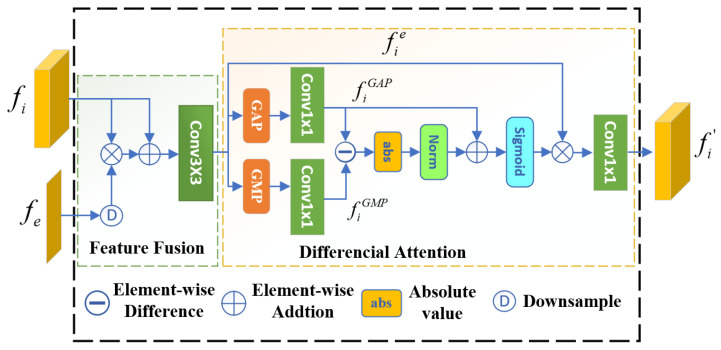
Architecture of the DAM module. In this module, the feature $f_{i}$ is first fused with the boundary prior $f_{e}$. The resulting features are then weighted using a differential attention mechanism, producing the enhanced feature $f_{i}^{\prime }$ that highlights the camouflaged object.

The architecture of DAM is shown in Figure 4. This module first integrates the boundary prior generated by the BDM with the features extracted by the backbone network. It then weights the fused features to highlight the camouflaged object using a differential attention mechinism. Accordingly, the workflow of DAM can be divided into two stages: boundary prior fusion and differential attention.

#### 3.3.1. Boundary Prior Fusion

**Figure 5:**
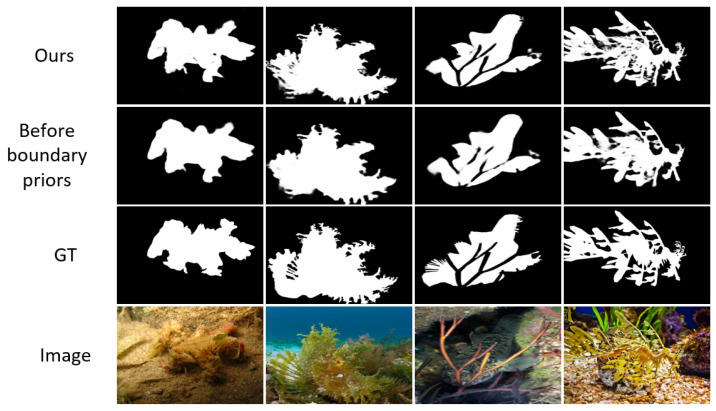
Comparison of visualization results from Boundary Prior Fusion.

To achieve the fusion, we multiply the boundary prior $f_{e}$ with the feature $f_{i}$ pixel by pixel, then add the result to the feature $f_{i}$ pixel by pixel. Finally, we apply a 3×3 convolution to the summed features, resulting in a preliminary boundary-guided feature $f_{i}^{e}$. It is important to note that $f_{i}$, extracted by the backbone network, can have four different possible sizes. Therefore, the boundary prior may need to be resized through downsampling before pixel-wise multiplication to ensure compatibility. As shown in the Figure 5, after applying the boundary prior fusion operation, the detail information in the feature visualization image is significantly improved. The entire process can be described by the following formula:

(3) $$f_{i}^{e}={Conv}_{3\times 3}\left(\left(f_{i}⊗D\left(f_{e}\right)\right)⊕f_{i}\right),\;i=\left(1,2,3,4\right)$$

where ⊕ denotes element-wise addition and D(·) represents downsampling.

#### 3.3.2. Differential Attention Mechanism

**Figure 6:**
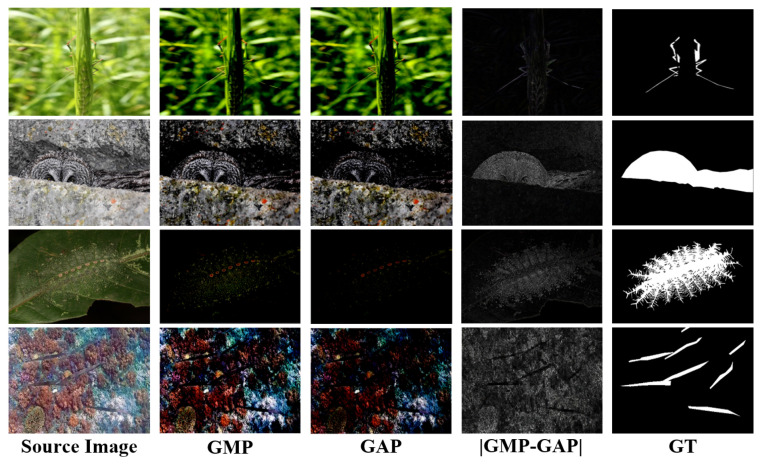
Comparison of the resulting images after applying GAP, GMP, and |GAP−GMP| on the source image.

To design the differential attention mechanism, we primarily leverage GAP and GMP. Guided by boundary priors, background features are suppressed by exploiting the difference between GAP and GMP.As illustrated in Figure 6, the absolute difference |GMP-GAP| demonstrates superior detection performance. Similar to AP and MP, GAP computes the average of all pixels in each channel, which tends to capture holistic features. In contrast, GMP selects the maximum pixel value from each channel, making it more suitable for highlighting the most prominent parts of the feature map. The difference between the two can suppress background information while highlighting the camouflaged object. Based on this observation, the feature map $f_{e}$ is processed in three parallel branches. Two branches apply GAP and GMP, respectively, and then subtract the results. The resulting difference is normalized and mapped to the range of (0,1) using the Sigmoid function. This output is used to weight the third branch, thereby enhancing the camouflaged target. The process can be described by the following formula:

(4) $$f_{i}^{\prime }=f_{i}^{e}⊗\sigma \left(Norm(abs\left(f_{i}^{\mathrm{GAP}}⊖f_{i}^{\mathrm{GMP}}\right)⊕f_{i}^{\mathrm{GAP}})\right).$$

Here, *i* represents the index of the feature layer, while abs(·) and Norm(·) denote the absolute value and normalization functions, respectively. Additionally, $f_{i}^{\mathrm{GAP}}$ and $f_{i}^{\mathrm{GMP}}$ represent the features obtained by applying GAP and GMP to $f_{e}$, followed by a 1×1 convolution.

### 3.4. Context Aggregation Module

The CAM module is designed to aggregate contextual information from each feature layer and generate the corresponding camouflage object mask. In this process, the CAM takes two inputs: the current layer’s features enhanced by the DAM and the mask output from the previous layer’s CAM. It is important to note that as the feature layer increases, the semantic information becomes richer while the detail information decreases, making mask prediction more challenging. Therefore, we generate masks only for the lower three layers. For the highest layer, $f_{4}^{\prime }$, it is combined with $f_{3}^{\prime }$ to generate the mask for layer 3. The implementation of CAM follows the method described in [6].

### 3.5. Loss Function

Our method produces four prediction results: three camouflaged object masks and one object edge. For each mask, we use a weighted binary cross-entropy loss $L_{BCE}^{\omega }$ and a weighted IOU loss $L_{IOU}^{\omega }$ [22] together to more accurately capture the key pixels in the image. For target edge learning, once the object’s spatial localization is obtained through multi-level feature fusion and refinement via the BDM, the edge mask is optimized using the Dice loss function. The Dice loss indirectly enhances boundary precision by maximizing the Dice similarity coefficient between the predicted mask and the ground truth. As a region-level metric, it is particularly sensitive to pixel discrepancies along object boundaries. During backpropagation, misclassifications at edge points produce large gradient updates, strongly guiding parameter optimization and enabling the model to generate segmentation results with sharper and more accurate contours. Consequently, the total loss function proposed by our method, denoted as $L_{\mathrm{Total}}$, can be formulated as:

(5) $$\begin{array}{c} L_{\mathrm{Total}}=\sum_{i=1}^{3}\left(L_{\mathrm{BCE}}^{\omega }\left(m_{i},G_{m}\right)+L_{\mathrm{IOU}}^{\omega }\left(m_{i},G_{m}\right)\right)\\ +\lambda L_{\mathrm{Dice}}\left(f_{e},G_{e}\right). \end{array}$$

Here, $G_{m}$ and $G_{e}$ represent the Ground Truth (GT) for the camouflaged object’s mask and edges, respectively. Correspondingly, $m_{i}$ and $f_{e}$ denote the predicted results for the camouflaged object’s mask and edges produced by the proposed method, where i=(1,2,3) indicates the feature layer index. The hyperparameter λ is set to 3.

## 4. Experiments

In this section, we present the details of our implementation and comprehensively compare BDA-Net with the latest COD methods on three publicly available datasets using common evaluation metrics. Additionally, we conduct ablation studies to validate the effectiveness of the key modules in our proposed method.

### 4.1. Implementation Details

In BDA-Net, we used PVTv2 pre-trained on ImageNet-1k as the backbone. During the training phase, we resized the input images to 416×416×3 and employed the Adam optimizer with a batch size of 12, setting the number of iterations to 50. Additionally, the learning rate was initially set to $1\times 10^{-4}$ and decayed according to a poly learning strategy with an exponent of 0.9. Our model was trained on an NVIDIA RTX 3090 GPU (with 24 GB memory).

### 4.2. Datasets

We trained and evaluated our models on three public benchmark datasets: CAMO [23], COD10K [2] and NC4K [24]. CAMO contains 1250 camouflaged images across 8 categories, with 1000 images used for training and 250 for testing. COD10K is the largest COD dataset, comprising 5066 images, with 3040 allocated for training and 2026 for testing, spanning 5 main categories and 69 subcategories. NC4K, consisting of 4121 images collected from the Internet, is the largest COD test set available to date.

### 4.3. Evaluation Metrics

We employ four widely used evaluation metrics to assess the performance of our model: Structure-measure ($S_{\alpha }$) [25], Mean Absolute Error (MAE) [26], weighted F-measure ($F_{\beta }^{\omega }$) [27], and average E-measure ($E_{\Phi }$) [28].

### 4.4. Comparison with SOTA Methods

We compare the proposed method with 12 SOTA methods including PFNet [4], SINet-v2 [29], SegMar [30], ZoomNet [5], TPRNet [31], DTINet [32], PolarNet [33], MSCAF-Net [34], SARNet [35], FEDER [36], FSPNet [37], EANet [10].

#### 4.4.1. Quantitative Comparison

The test results of the proposed method compared with 12 state-of-the-art COD methods on the COD10K, CAMO, and NC4K datasets are shown in Table 1. As evident from the table, BDA-Net consistently outperforms all other methods across all three datasets. Specifically, on the COD10K dataset, our method shows enhancements of 1.38% in $S_{\alpha }$, 3.60% in $F_{\beta }^{\omega }$, and 0.64% in $E_{\Phi }$ compared to the second-best method, SARNet. On the CAMO dataset, our method outperforms the second-best method, MSCAF-Net, with improvements of 0.69% in $S_{\alpha }$, 1.81% in $F_{\beta }^{\omega }$, and 0.22% in $E_{\Phi }$. Additionally, on the NC4K dataset, our method achieves better results with increases of 0.56% in $S_{\alpha }$, 1.31% in $F_{\beta }^{\omega }$, and 0.21% in $E_{\Phi }$ relative to the second-best method, SARNet.

#### 4.4.2. Evaluation Curves of COD Methods

**Figure 7:**
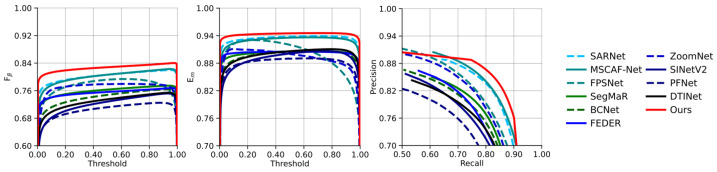
Presion-Recall, $F_{\beta }^{\omega }$-Threshold and $E_{\Phi }$-Threshold curves of BDA-Net and the recent SOTA algorithms on COD10K dataset.

To further evaluate the performance, we present the $F_{\beta }$-Threshold, $E_{m}$-Threshold, and Precision-Recall (PR) curves of BDA-Net compared with 12 other COD methods on the COD10K dataset, as shown in Figure 7, respectively. From the figures, it can be observed that BDA-Net consistently outperforms other methods in both the $F_{\beta }$-Threshold and $E_{m}$-Threshold curves, indicating higher detection accuracy. However, we also observe that when the recall rate of BDA-Net falls below 0.7, its precision slightly lags behind MSCAF-Net and SARNet, indicating that there is still room for further improvement.

BDA-Net delivers superior detection accuracy; however, this performance comes at the expense of reduced inference speed (3.67 FPS) and increased computational complexity, as reflected by a higher parameter count (69.476 M) and greater FLOPs (55.998 G). Therefore, BDA-Net is particularly well-suited for accuracy-critical applications, such as medical endoscopy, as well as latency-tolerant scenarios, including license plate recognition and underwater object detection.

### 4.5. Ablation Analysis

#### 4.5.1. Key Modules

Within BDA-Net, BDM and DAM are two key modules and represent our main contributions. To validate the effectiveness of the two modules, we conducted a series of ablation experiments, with the results shown in Table 2. It is worth noting that the baseline model retains only the PVTv2 backbone and CAM modules. To ensure consistency and feasibility across experiments, we employed identical experimental hardware and training parameters for all models—including the baseline model and variants utilizing BDM, DAM, and other architectures. Specifically, training was conducted on an NVIDIA RTX 3090 GPU (with 24 GB memory) using batch size 12 and hyperparameters set to 50 epochs.

From this table, we can observe at least two points. First, when adding either the BDM or DAM to the Baseline, the performance of the network improves to some extent. Specifically, there are obvious improvements in metrics $S_{\alpha }$ and $F_{\beta }^{\omega }$, while MAE and $E_{\Phi }$ remain largely unchanged. This indicates that enhancing the features extracted by the backbone network, whether through boundary detection or feature highlighting, contributes to improving the accuracy of camouflaged object detection. Second, when both BDM and DAM are added to the baseline, the network’s performance improves further, with all four metrics surpassing those of the baseline. This demonstrates that under the guidance of boundary information provided by BDM, DAM can more accurately highlight the features of camouflaged objects, thereby further enhancing detection performance.

**Figure 8:**
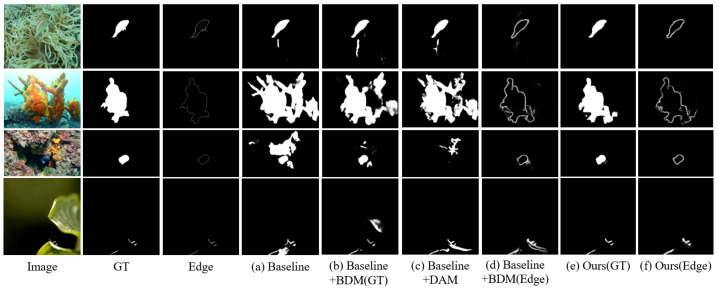
The visual comparison of detection results obtained by different models in the ablation study, (**a**) Baseline, (**b**) Baseline + BDM (GT), (**c**) Baseline + DAM, (**d**) Baseline + BDM (Edge), (**e**) Ours (GT), (**f**) ours (Edge).

To visually observe the impact of BDM and DAM on the detection results, we present the outputs of these four models on four test samples, as shown in Figure 8. From this figure, it can be seen that when using only the Baseline, the resulting masks contain not only camouflaged objects but also some background objects. However, when BDM is added to the Baseline, the resulting masks show a reduction in background objects, while the camouflaged objects remain unchanged. Conversely, when DAM is added to the Baseline, the resulting masks retain only high-contrast objects, and some or all of the camouflaged objects may be lost. Furthermore, when both BDM and DAM are added to the Baseline, the background objects in the resulting masks almost completely disappear, and the resulting masks become highly similar to GT. This indicates that, under the guidance of boundary information, DAM effectively focuses on the camouflaged object, thereby improving the accuracy of the resulting masks.

An insufficient number of input feature channels constrains the representational capacity of the model, often resulting in underfitting and the loss of discriminative information. Conversely, excessively large channel dimensions increase computational overhead and memory consumption while introducing redundant representations that may hinder generalization. To systematically examine this trade-off, we conducted an ablation study on channel dimension adjustment using 1 × 1 convolutional up-sampling within the BDM module. As summarized in Table 3, four configurations were evaluated: (N1) direct fusion of multi-level backbone features while preserving their original channel dimensions; (N2) unification of all feature layers to 16 channels; (N3) unification to 128 channels; and (N4) unification to 64 channels. The quantitative results indicate that the model achieves its best performance when the channel dimensions of all feature layers are consistent with those of $f_{1}$, suggesting that balanced channel allocation effectively preserves feature integrity while maintaining computational efficiency.

#### 4.5.2. Differential Attention Analysis

Within the DAM, the differential attention is achieved by performing ⊖ and ⊕ operations on the outputs of GAP and GMP, as shown in Equation (4). There are six possible combinations depending on the presence or absence of GAP and GMP, each representing a different attention mechanism. For simplicity, we label them as A1, A2, ⋯, A6. We tested these attention mechanisms on the COD10K dataset, and the results are presented in Table 4.

From Table 4, at least two key points can be observed: first, using only the operator ⊖, corresponding to A1, A3, and A5, yields $S_{\alpha }$ values of 0.873, 0.875, and 0.874, respectively, effectively highlighting camouflaged objects. This confirms the effectiveness of the attention mechanism. Second, the subsequent operator ⊕, corresponding to A2, A4, and A6, further improves attention to camouflaged objects, with $S_{\alpha }$ values of 0.876, 0.876, and 0.877, respectively. This indicates that operator ⊕ strengthens the effect of operator ⊖, and that the absolute difference |GAP−GMP| demonstrates better attention to camouflaged objects, leading to improved detection accuracy.

### 4.6. Analysis of the Effects of Different λ Hyparameters

To validate the effectiveness of λ hyperparameters in BDA-Net, experiments were conducted with different lambda settings. As shown in Table 5, the proposed loss function consistently enhances detection accuracy on the COD10K dataset.

### 4.7. Failure Cases

**Figure 9:**
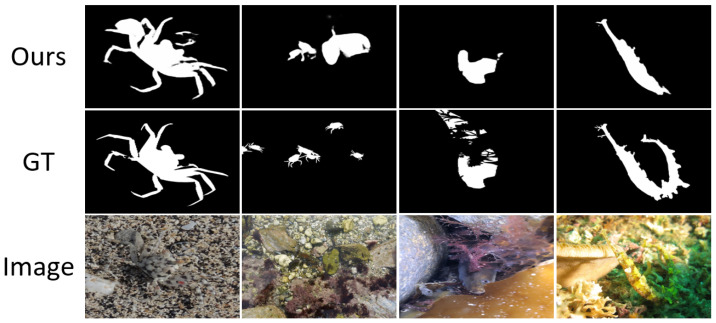
Visualization of detection failures in BDA-Net under severe conditions.

BDA-Net may exhibit biased predictions under challenging conditions. As shown in Figure 9, limitations arise in three scenarios: (1) objects with complex textures, where spatial reasoning is insufficient; (2) small targets, where discriminative features are inadequately captured; and (3) occluded objects, which the network often fails to identify. Future work will focus on improving robustness through enhanced camouflage modeling and feature representation learning.

## 5. Conclusions

Motivated by the human visual system’s strategy for detecting camouflaged objects—a process of progressive refinement from global contour localization to internal detail analysis—we introduce a BDA-Net for COD. In this network, we first extract boundary priors based on the multi-scale features of the image. Then, guided by the boundary information, we use the difference between GAP and GMP to suppress background features and construct a differential attention mechanism to highlight camouflaged objects. Finally, feature fusion is performed to achieve the segmentation of camouflaged objects. We evaluated BDA-Net on three commonly used COD datasets, and the results show that it delivers highly competitive performance compared to 12 state-of-the-art COD methods.

In subsequent investigations, our efforts will be directed toward addressing persistent challenges in COD, with particular emphasis on the accurate identification of microscale objects and partially occluded targets within complex natural scenes. These factors represent critical bottlenecks that currently impede high-precision detection performance. The development of robust computational mechanisms capable of effectively mitigating these issues will be instrumental in advancing BDA-Net’s operational robustness and real-world applicability under challenging environmental conditions.

## Figures and Tables

**Table 1:** Quantitative comparison with state-of-the-art methods for COD on three benchmarks using four widely used evaluation metrics ($S_{\alpha }$, MAE, $F_{\beta }$, $E_{\Phi }$).

Method	Year	COD10K				CAMO				NC4K			
		$S_{\alpha }↑$	MAE ↓	$F_{\beta }^{\omega }↑$	$E_{\Phi }↑$	$S_{\alpha }↑$	MAE ↓	$F_{\beta }^{\omega }↑$	$E_{\Phi }↑$	$S_{\alpha }↑$	MAE ↓	$F_{\beta }^{\omega }↑$	$E_{\Phi }↑$
PFNet [4]	2021	0.800	0.040	0.660	0.877	0.782	0.085	0.695	0.855	0.829	0.053	0.745	0.887
SINetV2 [29]	2022	0.815	0.037	0.680	0.887	0.820	0.070	0.743	0.882	0.847	0.048	0.770	0.903
SegMar [30]	2022	0.833	0.034	0.724	0.899	0.815	0.071	0.753	0.874	0.841	0.046	0.781	0.896
ZoomNet [5]	2022	0.838	0.029	0.729	0.888	0.820	0.066	0.752	0.877	0.853	0.043	0.784	0.896
DTINet [32]	2022	0.824	0.034	0.695	0.896	0.856	0.050	0.796	0.916	0.863	0.041	0.792	0.917
PolarNet [33]	2023	0.820	0.034	0.735	0.896	0.816	0.073	0.785	0.874	0.849	0.046	0.810	0.905
FEDER [36]	2023	0.822	0.032	0.716	0.900	0.802	0.071	0.738	0.867	0.847	0.044	0.789	0.907
BCNet [7]	2023	0.827	0.033	0.704	0.894	0.829	0.068	0.761	0.889	0.857	0.043	0.788	0.910
FSPNet [37]	2023	0.851	0.026	0.735	0.895	0.856	0.071	0.799	0.899	0.879	0.035	0.816	0.915
MSCAF-Net [34]	2023	0.865	0.024	0.775	0.927	0.873	0.046	0.828	0.929	0.887	0.032	0.838	0.934
SARNet [35]	2023	0.864	0.024	0.777	0.931	0.868	0.047	0.828	0.927	0.886	0.032	0.842	0.937
EANet [10]	2024	0.825	0.029	0.709	0.910	0.841	0.051	0.793	0.919	0.825	0.039	0.798	0.922
SAE-Net [38]	2024	0.837	0.064	0.770	0.891	0.837	0.064	0.770	0.891	0.862	0.042	0.796	0.912
PRNet [39]	2024	0.821	0.035	0.742	0.895	0.816	0.072	0.791	0.875	0.844	0.047	0.813	0.903
DSNet [40]	2025	0.809	0.038	0.657	0.878	0.817	0.073	0.726	0.870	0.843	0.050	0.753	0.894
**BDA-Net (Ours)**	-	**0.877**	**0.021**	**0.805**	**0.938**	**0.879**	**0.043**	**0.847**	**0.931**	**0.891**	**0.030**	**0.853**	**0.939**

**Table 2:** Quantitative evaluation for ablation studies on COD10K using four widely used evaluation metrics ($S_{\alpha }$, MAE, $F_{\beta }$, $E_{\Phi }$).

Model	COD10K			
	$S_{\alpha }↑$	MAE ↓	$F_{\beta }^{\omega }↑$	$E_{\Phi }↑$
Baseline	0.869	0.023	0.784	0.928
+BDM	0.874	0.023	0.796	0.928
+DAM	0.874	0.022	0.796	0.926
+DAM +BDM (Ours)	**0.877**	**0.021**	**0.805**	**0.938**

**Table 3:** Quantitative performance comparison of BDA-Net across different channel inputs on the COD10K dataset. The best results for each evaluation metric are marked in bold.

Model	COD10K			
	$S_{\alpha }↑$	MAE ↓	$F_{\beta }^{\omega }↑$	$E_{\Phi }↑$
N1	0.874	0.022	0.802	0.936
N2	0.873	0.022	0.802	0.934
N3	0.876	**0.021**	0.803	**0.938**
N4 (Ours)	**0.877**	**0.021**	**0.805**	**0.938**

**Table 4:** Quantitative evaluation for ablation studies of feature difference highlighting module on COD10K.

Attention	Combinations			COD10K			
	⊖		⊕				
	GMP	GAP	GAP	$S_{\alpha }↑$	MAE ↓	$F_{\beta }^{\omega }↑$	$E_{\Phi }↑$
A1	✓			0.873	0.022	0.794	0.933
A2	✓		✓	0.876	0.022	0.799	0.936
A3		✓		0.875	0.021	0.800	**0.938**
A4		✓	✓	0.876	0.021	0.799	0.937
A5	✓	✓		0.874	0.022	0.796	0.936
A6 (Ours)	✓	✓	✓	**0.877**	**0.021**	**0.805**	**0.938**

**Table 5:** Quantitative performance comparison of BDA-Net across different λ hyparameters inputs on the COD10K dataset. The best results for each evaluation metric are marked in bold.

Model	COD10K			
	$S_{\alpha }↑$	MAE ↓	$F_{\beta }^{\omega }↑$	$E_{\Phi }↑$
λ=1	0.874	0.023	0.799	0.933
λ=2	0.875	0.023	0.801	0.936
λ=3 (Ours)	**0.877**	**0.021**	**0.805**	**0.938**

## Data Availability

The raw data supporting the conclusions of this article will be made available by the authors on request.
